# Arsenic in Skin Diseases

**Published:** 1886-08

**Authors:** 


### *ARSENIC IN SKIN DISEASES.*

The editor of the *Journal of Cutaneous and Venereal Diseases* is desirous of ascertaining to what extent arsenic is used by American physicians in the treatment of skin diseases, and also the result of their experience as to its therapeutical value.

Information upon the following points is requested of every physician who reads this :

Are you in the habit of employing arsenic, *generally*, in the treatment of skin diseases ?

In what diseases of the skin have you found arsenic of superior value to other remedies ?

What ill effects, if any, have you observed from its use ?

What preparation of the drug do you prefer, and in what doses do you employ it ?

Address, Editor of Journal of Cutaneous and Venereal Diseases, 66 West 40th Street, New York.

---

MAURICE HACHE, M. D., 8 Rue de Tournon, Paris, May 18, 1886, says: I have tried Bromidia in two cases, one patient suffering from a slight febrile affection, the other a victim of acute insomnia; in the latter case, various preparations of opium had proved useless, and the administration of chloral was followed by lassitude and congestion in the head. Bromidia produced sound sleep in both of these cases, unaccompanied by any unpleasantness on awaking. In my opinion, this preparation is destined to render good service, and I intend prescribing it whenever the opportunity presents itself.

